# Mapping mosquito diversity in Kenya correlating species distribution with malaria prevalence across varied climatic parameters

**DOI:** 10.1186/s12936-025-05713-y

**Published:** 2026-01-05

**Authors:** Jeremiah O. Zablon, Varun Goel, David Giesbrecht, Lenson Kariuki, Charles Mbogo, William C. Goedel, Jeffrey A. Bailey, Damaris Matoke-Muhia

**Affiliations:** 1 School of Public Health, Brown University, Providence, RI USA; 2 Department of Pathology and Laboratory Medicine, Brown University, Providence, RI USA; 3 Centre for Biotechnology Research and Development, Kenya Medical Research Institute, Nairobi, Kenya; 4 Department of Geography, University of South Carolina, Columbia, SC USA; 5 Division of National Malaria Programme, Ministry of Health, Nairobi, Kenya; 6 Center for Geographic Medicine Research, Coast, Kenya Medical Research Institute, Kilifi, Kenya; 7 Center for Computational Molecular Biology, Brown University, Providence, RI USA

**Keywords:** Malaria, *Anopheles arabiensis*, *Anopheles coluzzii*, *Anopheles gambiae*, *Anopheles funestus*, *Anopheles moucheti*, *Anopheles nili*, Climate factor

## Abstract

**Background:**

Malaria is a major public health problem, with over half of the world’s population, primarily in sub-Saharan Africa, at risk. Climate change is already affecting the prevalence of malaria, in part by altering the distribution and density of mosquito vectors. This study assessed the relationship between climate variables and mosquito species distribution on malaria prevalence in Kenya from January 2019 to June 2021.

**Methods:**

Data from 23 Kenyan counties were analyzed using mixed-effect regression, spatial analysis, correlation analysis, and the Least Absolute Shrinkage and Selection Operator (LASSO) to select variables.

**Results:**

Malaria prevalence was highest in the Lake Endemic (19%), followed by the Coastal Endemic (5%), semi-arid seasonal (2%), and low-risk (0.9%) malaria epidemiologic zones in 2020. Mosquito species distribution varied, with distinct ecological preferences observed with *Anopheles gambiae* dominated coastal and semi-arid areas, while *Anopheles funestus* was highest in the highland and lake zones. Regression analysis indicated that a combination of environmental factors and public health interventions, including insecticide-treated nets (ITN) coverage, was an important predictor of malaria prevalence. However, the relationship between these factors and malaria prevalence varied across regions and time periods.

**Conclusion:**

This study provides insights into the spatial dynamics of mosquito species and their distribution in relation to malaria epidemiological zones in Kenya, thereby informing targeted control strategies.

**Supplementary Information:**

The online version contains supplementary material available at 10.1186/s12936-025-05713-y.

## Background

Malaria is one of the most significant public health problems globally; the World Malaria Report of 2021 estimated 627,000 deaths, and 241 million malaria cases were reported in 2020 [1]. New malaria cases have increased to 263 million in 2025, and hence, more than half the world’s population is still at risk of malaria, with endemicity in 100 countries [2]. African countries suffer the greatest burden of malaria, with 92% of malaria-related deaths and 90% of all global malaria cases [3]. In Kenya, malaria is a major public health problem with approximately 70% of the population at risk. An estimated four million cases are reported yearly, with a 5.1% mortality rate among patients with severe malaria [4].

Malaria occurs through the bite of *Plasmodium*-infected female mosquitoes of the *Anopheles* genus [5]. The African continent is home to at least 140 *Anopheles* species, of which 6 species (*Anopheles arabiensis*, *Anopheles gambiae*, *Anopheles moucheti, Anopheles funestus*, *Anopheles nili*, and *Anopheles coluzzii)* account for 95% of all human malaria transmission [6]. In Kenya, the key *Anopheles* mosquito species responsible for transmitting malaria include *An. arabiensis*, *An. gambiae*, *Anopheles merus*, and *An. Funestus *[7, 8]*.* The primary cause of malaria in sub-Saharan Africa is *Plasmodium falciparum,* which accounts for at least 99.7% of all malaria cases [6, 7]. However, other malarial parasites, such as *Plasmodium ovale* and *Plasmodium malariae,* account for the remaining proportion of malaria cases in this region and many subclinical infections [7].

Kenya has been classified into four malaria epidemiological zones according to the Kenya Malaria Indicator Survey (KMIS). The zones were defined based on malaria transmission levels, climatic factors, and topography [4, 5, 9]. The seasonally arid zone in the northern and southern parts of the country is characterized by dry climatic conditions and experiences short periods of intense malaria transmission during the rainy seasons [8, 9]. The low-risk zones, which include Nairobi and Central Kenya, are characterized as highland regions but experience cooler temperatures than other parts of Kenya’s highlands; hence, they have lower malaria transmission than the rest of the highlands [4, 7, 10].

There has been a significant reduction in malaria transmission reported in Uganda, particularly in Tororo District, due to the universal distribution of long-lasting insecticide-treated nets (LLINs) and sustained indoor residual spraying (IRS), with transmission intensity decreasing 500-fold between 2011 and 2019 [11]. However, a resurgence of malaria cases occurred following a change in IRS insecticide formulations in 2019–20, underscoring the challenges in sustaining long-term progress [9]. Building on the experience in Uganda in the resurgence of malaria cases, the changing dynamics of mosquito populations in Kenya also carry significant implications for malaria control. As climatic conditions and land-use practices continue to evolve, monitoring and adapting to these changes are essential for effective malaria prevention and control strategies. Public health initiatives must consider these factors to mitigate malaria’s impact and reduce transmission in affected regions.

Changes in temperature and the hydrological cycle will likely make conditions conducive to malaria transmission in these previously low-risk zones [4, 12]. In addition, high humidity levels are ideal for mosquito breeding, and rainfall can create breeding grounds for mosquitoes [10]. Climate change, particularly changes in temperature and precipitation, affects the survival, distribution, and abundance of mosquitoes, which can, in turn, affect malaria transmission [13]. An increase in temperature and rainfall can increase mosquito breeding and improve mosquito survival, leading to greater malaria transmission [12]. High temperatures alone can increase the incubation rate of the malaria parasite in the mosquito, leading to younger, more infective mosquitoes [1]. Higher temperatures may reduce the parasite’s extrinsic incubation period (EIP) to 7 days instead of 10–14 days; as a result, even younger mosquitoes may carry the parasite and infect humans, rather than primarily older mosquitoes. This means more mosquitoes can spread malaria in hotter conditions [9]. Model projections indicate that climate change will expand the geographical distribution of mosquito-borne diseases, leading to increased malaria transmission in regions previously considered low-risk [10]. This expansion is expected to include higher-altitude areas, where the proliferation of malaria vectors may facilitate transmission among populations with limited prior exposure [8].

There has been little effort to understand the distribution of female *Anopheles* mosquitoes in Kenya, leaving a critical gap in knowledge regarding current species distributions, the role of climatic factors, and the combined impacts of vector control on mosquito dynamics and malaria prevalence [4, 14]. To ensure vector control interventions are effective and well-targeted, accurate and up-to-date information is essential, especially in the face of emerging insecticide resistance and shifting environmental conditions. Strengthening research on the distribution of female *Anopheles* mosquitoes is, therefore, vital for designing sustainable, evidence-based, and region-specific malaria control strategies. This study aims to examine the spatial distribution of mosquito species across Kenya’s malaria epidemiological zones and to assess how climatic factors influence these patterns, generating evidence to guide targeted malaria control strategies.

## Methods

### Study design

This study used secondary data collected by the Kenya National Malaria Control Program (NMCP) under the revised malaria strategy, an ongoing surveillance program with approved study number KEMRI/RES/7/3/1 [1]. The study used routine vector surveillance data collected in all 23 counties between January 2019 and June 2021. In each county, three sub-counties were surveyed at the village level. Two villages and 14 households in each sub-county were sampled, and each household completed a survey collecting basic demographic and household data on malaria, including the number of ITNs. Mosquito collection in each household was done using an aspirator from 0600 to 0900 h by trained collectors. The collection was carried inside houses. The samples were transported to the Kenya national reference laboratory for molecular analysis (PCR) to confirm *Anopheles* species and ELISA to detect malaria parasite sporozoites.

Data were also sourced from the cross-sectional malaria indicator surveys conducted from 2019 to 2021 [9, 13, 15–18]. The surveys used stratified two-stage designs, with households selected from enumeration areas generated from census data. A standard Demographic Health Survey (DHS) questionnaire collected the demographic and malaria-related data, including follow-up from previous surveys (https://dhsprogram.com) [4]. Malaria data include insecticide ownership, malaria testing, access to and coverage of nets, malaria in pregnancy, haemoglobin, case management of fever in children, and prevalence of *Plasmodium* species, based on blood samples collected. Data were also drawn from the 2015 and 2023 DHS surveys, which comprised a nationally representative sample of 7952 households. The survey provided national malaria prevalence estimates for rural and urban areas in all four malaria epidemiological zones, counties, and sub-counties [19]. Sporozoite detection was conducted using enzyme-linked immunosorbent assay (ELISA) targeting the circulating sporozoite protein of *P. falciparum*, following standard WHO protocols. The proportion of mosquitoes testing positive for sporozoites was calculated as the sporozoite positivity rate [19, 20].

### Measures

Climate and environmental data were sourced from the University of East Anglia Climatic Research Institute (CRU) [21]. The primary exposures were climatic factors (temperature, precipitation, humidity, and rainfall) previously linked to malaria transmission. Although previous studies have demonstrated associations between climatic factors and malaria transmission in other settings, this evaluation adds value by contextualizing these relationships within the current Kenyan setting. Climatic factors were measured at the county level and summarized as monthly averages. Variables were then extracted from the village’s geographic location where the mosquitoes were collected. The download was processed to a high resolution of 0.5 × 0.5 degrees in longitude and latitude for the sampled villages. The GPS location used in the study was from the centre of the villages where mosquitoes were sampled to protect the privacy of the households where data were collected. Village distances were calculated using Thiessen polygons in ArcGIS Pro, with a minimum distance of 4 km and a maximum of 8 km [21]. Data from 2019 through 2021 were cleaned and used for analysis. The spatial distribution of mosquito species over time was also included, as they mediate the effects of climate on malaria transmission. Additionally, this study included the proportion of the population with protected insecticide-treated nets [22].

### Statistical analysis

Monthly malaria prevalence, trends, and climatic factors were computed as frequencies and percentages. Variance was determined to assess seasonal changes in malaria prevalence and climatic conditions using R (*version 4.2.1*) [23]. The Least Absolute Shrinkage and Selection Operator (LASSO) regression was employed to identify the variables with the strongest associations in the modelling analysis. A logistic mixed-effects regression model was used to estimate the relationship between the dependent variable (malaria prevalence) and one or more independent variables (as described above). The mixed-effects models account for variability across locations and allow the inclusion of random effects to capture unobserved heterogeneity in the data. A significant threshold of p < 0.05 was used for all analyses.

## Results

### Descriptive statistics

A total of 6054 *Anopheles* mosquitoes were collected, with *An. gambiae* comprising 73% (4406) and *An. funestus* accounting for 27% (1648). The distribution of species varied across epidemiological zones, as shown in Table 1.* Anopheles gambiae* dominated in the Coast Endemic (66.2%) and Semi-arid seasonal zones (63.8%), while the Highland epidemic zone showed the highest proportion of *An. funestus* (19.8%), accompanied by the highest sporozoite positivity (2.91%). The Lake endemic zone exhibited a more balanced species distribution, with *An. funestus* (64.5%) exceeding *An. gambiae* (35.5%). Sporozoite positivity varied across epidemiological zones, ranging from 0.81% in the Coast endemic zone to 2.91% in the Highland epidemic zone, with intermediate rates observed in the Lake endemic (1.48%) and Semi-arid seasonal zones (0.86%).

**Table 1:** Distribution of sporozoite positivity and *Anopheles* mosquito species across epidemiological zones

Malaria zone	*An. gambiae* (n, %)	*An. funestus* (n, %)	Total mosquitoes (n)	Sporozoite-positive (n)	Mosquitoes tested (n)	Sporozoite positivity (%)
Coast endemic	16,120 (66.2%)	8,216 (33.8%)	24,336	7	865	0.81
Highland epidemic	171,600 (80.2%)	42,224 (19.8%)	213,824	26	895	2.91
Lake endemic	13,104 (35.5%)	23,808 (64.5%)	36,912	16	1,083	1.48
Semi-arid seasonal	53,560 (63.8%)	30,471 (36.2%)	84,031	12	1,394	0.86

Malaria incidence in 2020 peaked in Turkana (0.413), Nandi (0.352), and Machakos (0.301), while counties such as Embu, Kirinyaga, Kitui, Meru, and Samburu recorded zero cases. Higher malaria prevalence rates corresponded with elevated temperatures and increased precipitation, particularly in counties such as Busia (38.5%), Siaya (23.8%), and Bungoma (23.2%).

Insecticide-Treated Nets (ITNs) usage among children varied considerably from as low as 25% in counties such as Baringo, Kitui, and Meru, to a high of 58% (95% CI 52.1%, 63.9%) in Siaya and Busia. Additionally, counties exhibited significant diversity; population density was highest in Kitui, while rainfall (between 1200 mm and 1800 mm) was particularly high in Siaya. These variations underscore the complex interplay among environmental, socioeconomic, and epidemiological factors that influence mosquito distribution and malaria dynamics. Counties with higher malaria prevalence had correspondingly higher temperatures and precipitation. For instance, in 2019, Busia and Siaya had higher malaria prevalence alongside increased temperatures and precipitation (Table 2), while in 2020, Bungoma, Busia, and Siaya had higher malaria prevalence (Fig. 2).

**Table 2:** Determinants of malaria risk: regression estimates for environmental and climatic variables in Kenya (2015 vs. 2020)

Variable	2020 Estimate standard error (SE)	2015 Estimate standard error (SE)
Aridity	− 0.0040*** (0.0010)	0.0003 (0.0010)
Elevation	0.0000 (0.0000)	0.0000** (0.0000)
Enhanced vegetation index	0.1780*** (0.0420)	0.0620 (0.0380)
Frost days	− 0.0350 (0.0460)	− 0.0150 (0.0320)
Irrigation	0.0004 (0.0010)	0.0010 (0.0010)
ITN coverage	−0.0700 (0.0440)	− 0.0050 (0.0150)
Maximum temperature	0.0100 (0.0200)	0.0010 (0.0200)
Nightlights composite	− 0.0010 (0.0010)	− 0.0060*** (0.0010)
Night land surface temp	0.0080*** (0.0020)	0.0060*** (0.0020)
Precipitation	0.0200*** (0.0040)	− 0.0002 (0.0030)
Rainfall	− 0.0000 (0.0000)	0.0000 (0.0000)
UN population density	0.0000 (0.0000)	0.0000 (0.0000)
Wet Days	0.0010 (0.0020)	0.0020 (0.0020)
Constant	− 0.2100* (0.0940)	− 0.1920*** (0.0620)
Observations	186	186
R^2^	0.527	0.367
Adjusted R^2^	0.491	0.319
Residual std. error	0.027 (df = 172)	0.021
F Statistic	14.730*** (df = 13; 172)	7.673***

Statistical significance codes are attached below

. → 0.05 p-value ≤ 0.1 (weak evidence, sometimes called “marginal significance”)

* → 0.01 p-value ≤ 0.05 (moderate evidence)

** → 0.001 p-value ≤ 0.01 (strong evidence)

*** → p-value ≤ 0.001 (very strong evidence against the null)

### Univariate associations between climatic parameters and malaria prevalence

**Fig. 1:**
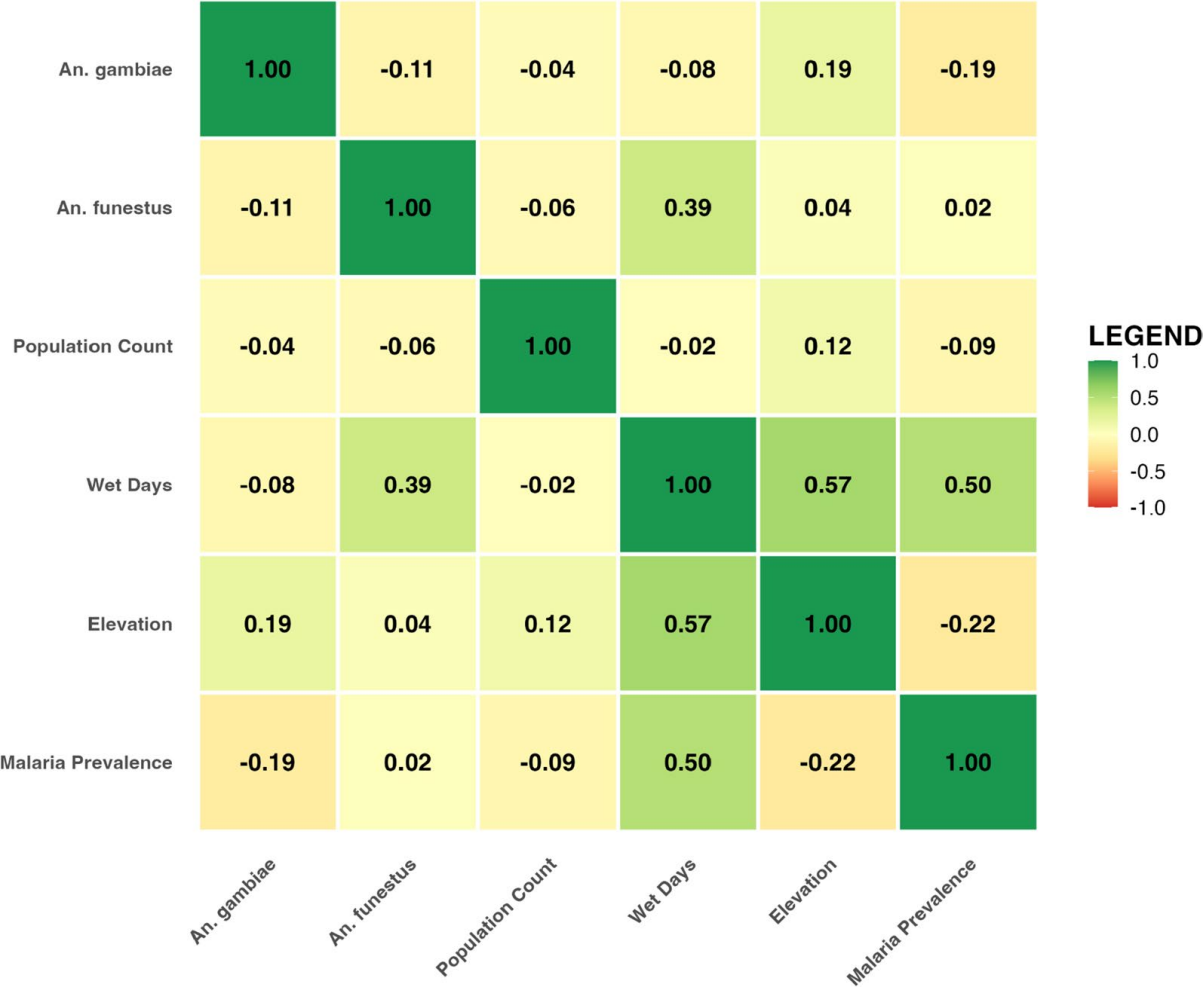
Correlation Matrix of Malaria Prevalence, Mosquito Abundance, and Environmental Factors by County in Kenya. The heatmap displays Pearson correlation coefficients between variables. Positive correlations are shown in green and negative correlations in red. The intensity of color in the color scale reflects the strength of the relationship

The correlation matrix shown in (Fig. 1) illustrates the relationships between malaria prevalence, mosquito abundance, and *An. gambiae* and *An. funestus*, and environmental variables, population count, wet days, and elevation across Kenyan counties. Notably, wet days show a moderate positive correlation with malaria prevalence (r = 0.50), suggesting a potential role for rainfall in creating breeding conditions for malaria vectors. Elevation is negatively correlated with malaria prevalence (r = − 0.22), suggesting that lower-altitude areas may potentially be more prone to malaria transmission. Associations varied by mosquito species. *Anopheles gambiae* displays a weak negative correlation with malaria prevalence (r = − 0.19), while *An. funestus* has a negligible correlation (r = 0.02), indicating possible regional or behavioral differences in transmission dynamics. Wet days and elevation also show a positive association (r = 0.57), reflecting potential climatic gradients. The correlation analysis underscores the complex interplay between environmental factors and disease dynamics, underscoring the need for geographically targeted malaria control strategies that account for ecological and entomological variability. Environmental factors (temperature, rainfall) strongly influence malaria incidence and mosquito populations. Human interventions (e.g., ITNs) and socioeconomic conditions (population density, household size) play crucial roles in malaria dynamics, potentially mitigating impacts despite the presence of mosquitoes.

**Fig. 2:**
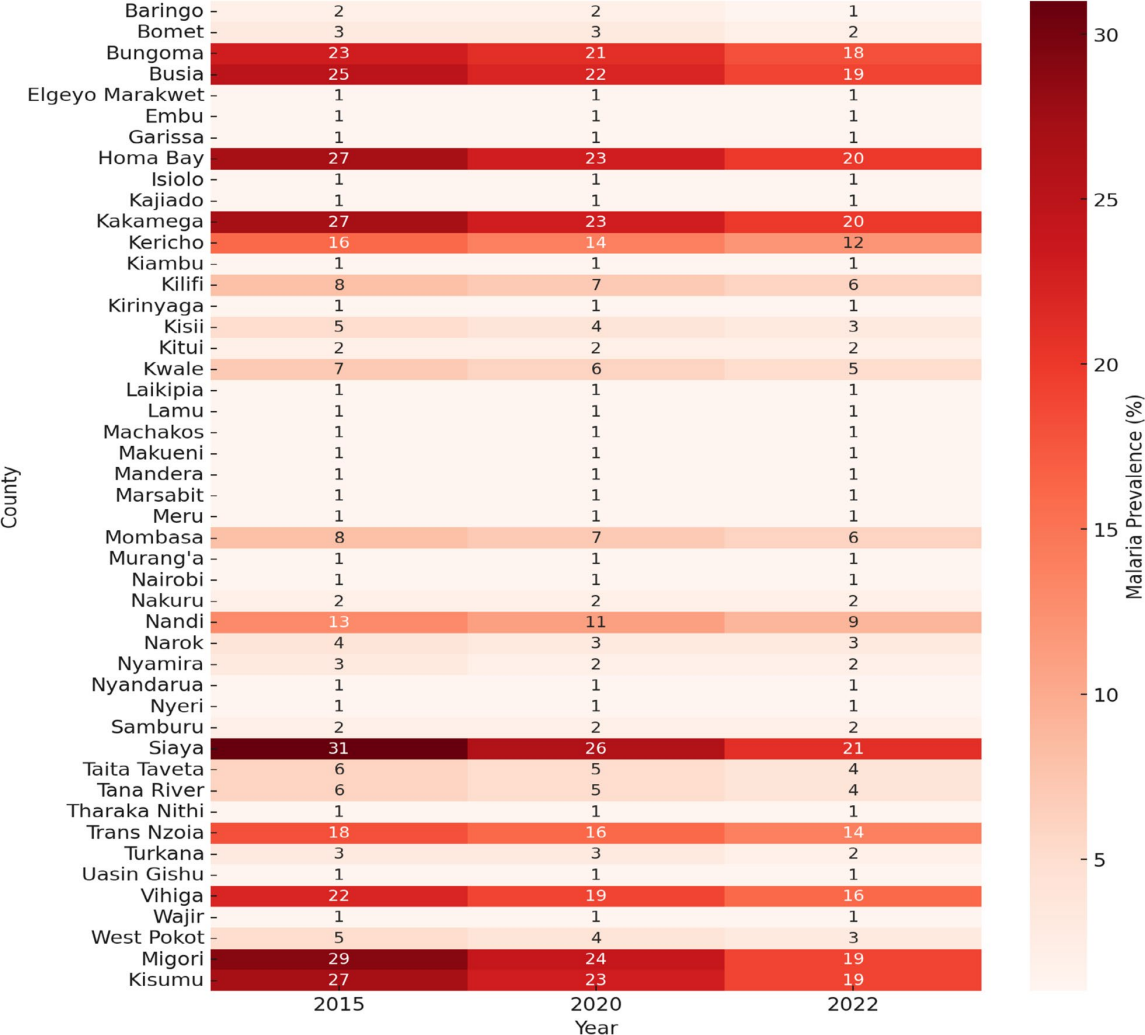
Temporal Trends in Malaria Prevalence Across Kenyan Counties between 2015 and 2022. The heatmap displays the county-level malaria percent (%) prevalence derived from the Kenya Malaria Indicator Surveys conducted in 2015, 2020, and 2022. Darker shades in the color scale indicate higher malaria prevalence

The heatmap (Fig. 2) illustrates malaria prevalence (%) across Kenyan counties from 2019 to 2023. Kakamega, Kisumu, Migori, Homa Bay, and Siaya are among the counties with the highest malaria burden. In 2020 and 2021, these regions experienced significant transmission, influenced by environmental, climatic, and socioeconomic factors, and possibly linked to seasonal patterns or the effectiveness of interventions. The county-level temporal variations highlight fluctuating transmission intensity, with persistent hotspots and areas of emerging concern. The data underscore the importance of targeted interventions based on county-specific malaria trends and environmental conditions.

### Mixed effect model

A mixed-effects regression model was employed to examine how environmental and socio-economic factors influenced malaria prevalence in Kenya over the five years from 2015 to 2020 (Table 2). The analysis reveals a stronger model fit in 2020, with an R^2^ of 0.527, compared to 0.367 in 2015. This indicates that the 2020 data better explain the variation in malaria prevalence.

Among the significant findings, aridity emerged as a key negative predictor in 2020 (β =  − 0.004, p < 0.01), suggesting that drier regions had lower malaria transmission, likely due to fewer breeding sites for mosquitoes. Greener environments, as measured by the Enhanced Vegetation Index (EVI), were positively associated with malaria prevalence in 2020 (β = 0.178, *p* < 0.01), suggesting that vegetation may support higher mosquito densities.

Temperature effects varied: nighttime land surface temperature showed a consistent, significant positive association with malaria prevalence across both years (2020: β = 0.008; 2015: β = 0.006; both *p* < 0.01), emphasizing the role of warm nocturnal conditions in facilitating mosquito activity and parasite development. In contrast, maximum daytime temperature was not a significant predictor in either year.

Precipitation significantly increased malaria risk in 2020 (β = 0.0200, SE = 0.0040, *p* < 0.01) but had no significant effect in 2015 (β =  − 0.0002, SE = 0.0030). Interestingly, rainfall did not show a statistically significant association with malaria prevalence in either year (2020: β =  − 0.0000, SE = 0.0000; 2015: β = 0.0000, SE = 0.0000), suggesting that other hydrological or environmental factors may play a more prominent role.

### Spatial distribution of mosquito species across different epidemiological zones

**Fig. 3:**
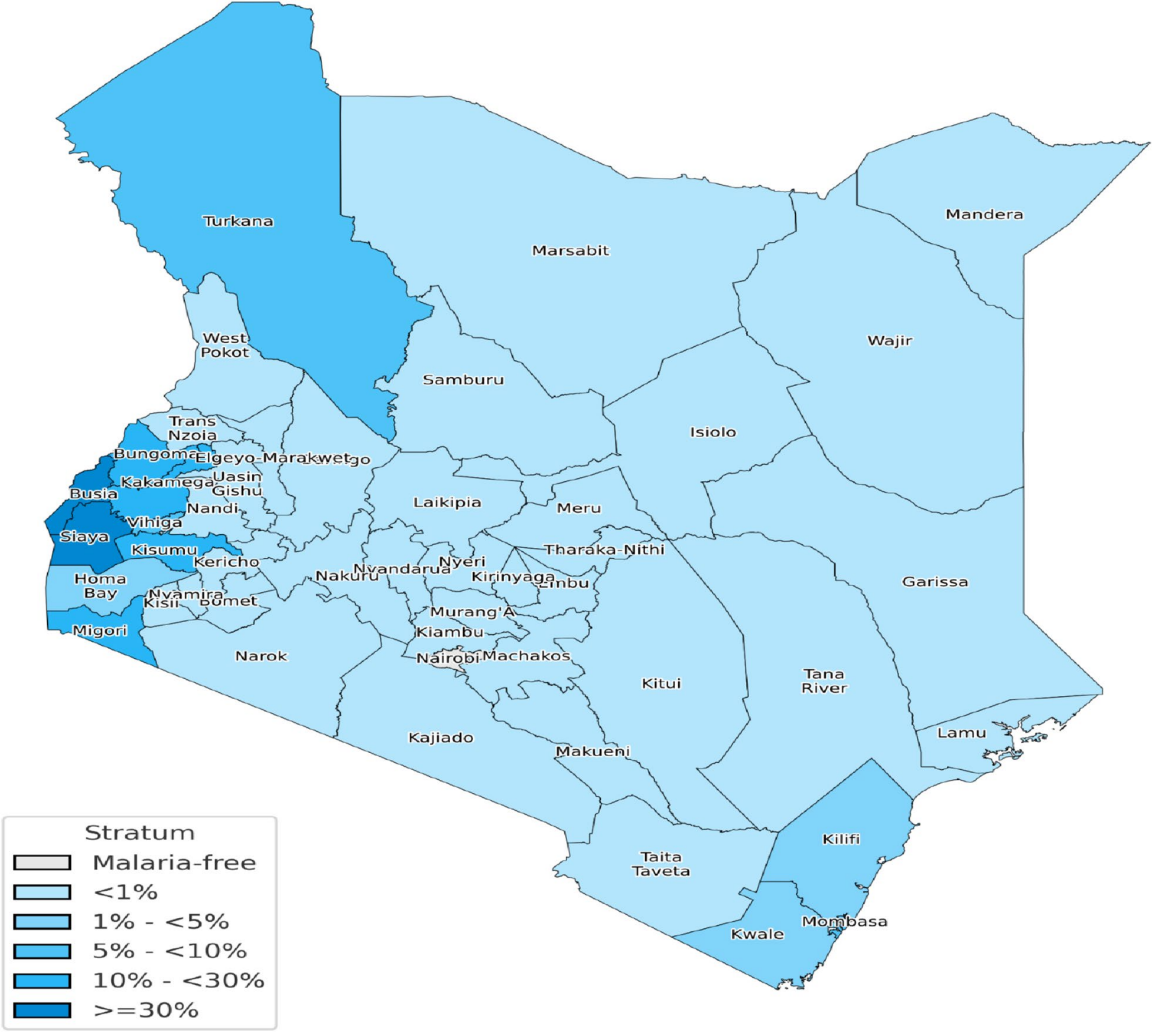
Spatial distribution of malaria infection risk zones across Kenya. The map represents malaria transmission strata classified by infection risk levels ranging from malaria-free to high-risk zones (0– > 30% prevalence). The data was extracted from the Ministry of Health, National Malaria Control Program (2023)

The choropleth map (Fig. 3) illustrates the malaria epidemiological zones across Kenya, classified by transmission risk. These zones are defined based on a combination of altitude and climatic conditions, including temperature, rainfall, humidity, and malaria transmission intensity. Lake Endemic zones, such as those around the Lake Victoria basin and western Kenya, experience the highest year-round transmission due to warm temperatures and high humidity. Highland regions are epidemic-prone, having seasonal outbreaks driven by fluctuating rainfall and lower population immunity. Arid and Semi-Arid regions exhibit seasonal transmission, with malaria peaking during rainy periods. In contrast, Highland Epidemic zones covering central Kenya and some high-altitude areas are considered low risk, as cooler temperatures and ecological factors limit mosquito survival and parasite development. Figure 3 shows that about 70% of Kenya’s population lives in moderate-to-high transmission areas, underscoring the need for targeted malaria control strategies and efficient resource allocation.

**Fig. 4:**
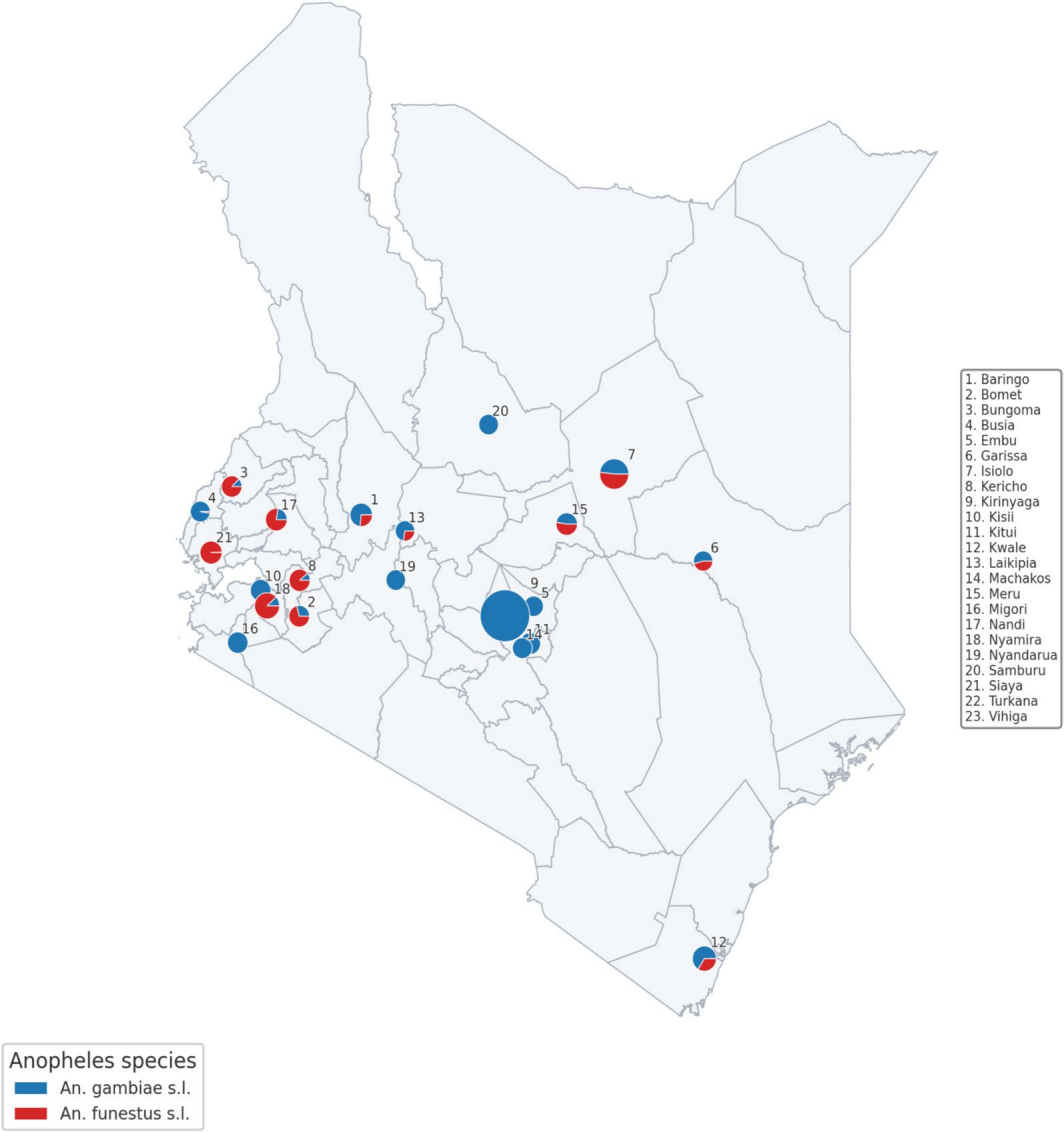
A map of Kenya showing the distribution of *An. gambiae s.l.* and *An. funestus s.l. in* Kenya from 2019 to 2023. Proportional pie charts represent relative abundances of *An. gambiae s.l.* and *An. funestus* s*.l.* based on aggregated mosquito surveillance data. Counties are numbered on the map, with corresponding names provided in the legend

Distinct ecological preferences were observed with *An. gambiae* is most abundant in regions like Kerugoya and Embu, while *An. funestus* dominated in Isiolo, Kisii, and Nyamira (Fig. 4). These variations align with each species’ environmental requirements and reflect local breeding site availability. Despite high mosquito abundance in some areas, malaria prevalence did not align consistently. County-level regression and correlation analyses revealed a modest negative correlation between *An. gambiae* and malaria prevalence (r = − 0.24) and a weak positive correlation for *An. funestus* (r = 0.12). The two species were slightly negatively correlated (r = − 0.12), suggesting they may occupy different ecological niches or respond to varying local conditions.

Linear regression models further showed that neither species significantly predicted malaria prevalence. *Anopheles gambiae* had a non-significant negative coefficient (β = − 3.09e-06, *p* = 0.29, R^2^ = 0.058), and *An. funestus* had a weak, non-significant positive coefficient (β = 3.64e-06, *p* = 0.62, R^2^ = 0.013). This suggests that mosquito counts alone do not adequately capture the complexity of malaria transmission. County-specific analysis showed interesting contrasts. Kirinyaga, with the highest *An. gambiae* count reported zero malaria prevalence, possibly due to effective control measures or data gaps. Conversely, counties like Nandi, Kericho, and Bungoma showed both high *An. funestus* counts and elevated malaria prevalence, suggesting a stronger role for *An. funestus* in sustaining transmission.

**Fig. 5:**
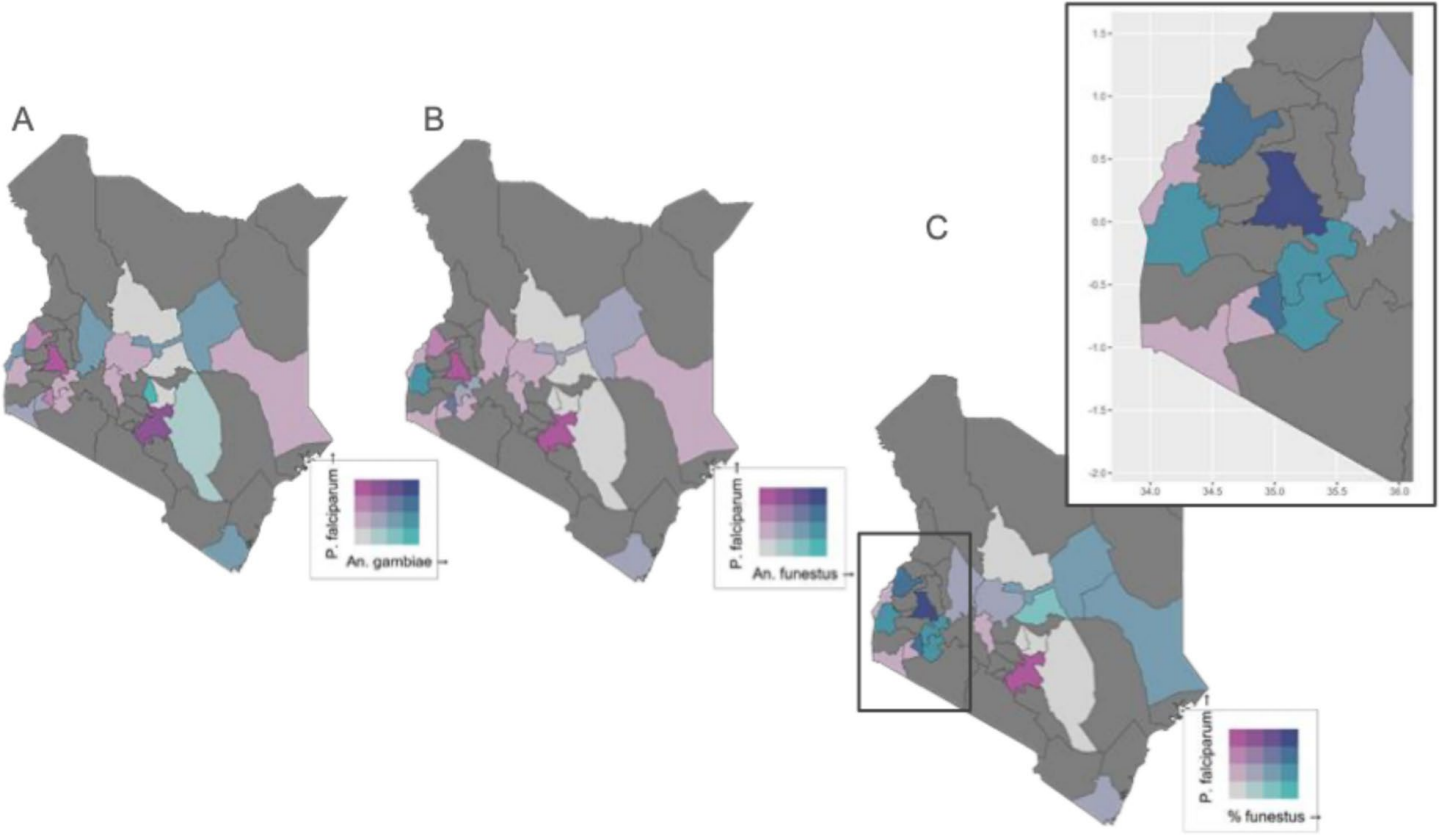
Spatial interaction between *Plasmodium falciparum* prevalence and mosquito vector abundance in Kenya. Bivariate maps illustrate the geographic distribution and interaction of *P. falciparum* prevalence with relative abundance of key malaria vectors; **A** *An. gambiae*, **B** *An. funestus*. **C** represents a risk synthesis map of the relative abundance of *P. falciparum* and *An. funestus* to highlight high risk areas based on high parasite prevalence and vector abundance. Colour gradients represent the interaction between parasite prevalence (pink) and vector abundance (blue). The bivariate legend in each panel reflects the intensity of co-occurrence, with darker hues representing higher combined risk

The analysis of malaria prevalence and mosquito species distribution in Kenya reveals critical insights into the spatial dynamics of malaria transmission across various epidemiological zones. Figure 5 highlights that malaria incidence is strongly associated with two mosquito species, *An. funestus* and *An. gambiae*. This association is particularly strong in Turkana County and moderately strong in Machakos, Bungoma, and Siaya counties. Overall, Kenya is divided into four malaria epidemiological zones.: Lake Endemic, Semi-Arid Seasonal, Highland Epidemic, and Coast Endemic. Both mosquito species are present across all zones; however, notable regional patterns emerge.

The Lake Endemic zone records the highest abundance of *An. funestus*, whereas the Coast Endemic zone exhibits a higher presence of *An. gambiae*. As shown in Fig. 5, counties in western Kenya have high *An*. *funestus* abundance and high *P. falciparum* prevalence, supporting the hypothesis that *Infests* is driving transmission in these counties. An emphasis on region-specific treatments is necessary in the western counties with high *An. funestus* abundance since they also have high malaria prevalence. Precisely, Fig. 5A shows strong co-occurrence of *P. falciparum* and *An. gambiae* in western Kenya, while Fig. 5B highlights more localized overlap between *P. falciparum* and *An. funestus*, particularly in western and coastal regions. Figure 5C synthesizes these patterns to identify the western parts of Kenya (Lake Endemic Zone) as high-risk areas where both parasite prevalence and *An. funestus* abundance is elevated. The data underscore the importance of targeted interventions based on county-specific malaria trends and environmental conditions.

## Discussion

The purpose of this study was to investigate the distribution of female *Anopheles* mosquitoes in Kenya and to evaluate the impact of climate on their role in malaria transmission across various epidemiological zones. There are still gaps in our knowledge of species dynamics, particularly in understanding how changing climatic and environmental conditions emerge, despite continuous efforts to control malaria. Through combining entomological surveys with village-level climate data, this study offers up-to-date information on sporozoite positivity and mosquito species distribution. The results are intended to improve focused malaria control, guide adaptive measures, and address problems caused by changing malaria transmission patterns in Kenya, climate change, and pesticide resistance.

The geographical distribution of *Anopheles* mosquitoes in Kenya is significantly influenced by various climatic factors, such as humidity, rainfall, and temperature [3, 25]. The distribution of mosquito species varied by epidemiological zone, with *An. gambiae* is more dominant in the Semi-Arid Seasonal and Coast Endemic zones, while *An. funestus* was prevalent in the Lake Endemic zone and Highland Epidemic zones. The study findings align with those of a study conducted in Tanzania, which indicates species’ adaptability to changing environments [24]. Incorporating statistical significance testing and mapping these results against rainfall and land cover will strengthen vector control strategies [20, 26].

The correlation analysis revealed that precipitation, rainfall, and the enhanced vegetation index (EVI) were positively correlated with malaria prevalence, whereas aridity and maximum temperature were negatively correlated with it. The mixed effects model also identified population count and aridity as significant predictors of malaria prevalence (p < 0.05).

Based on 2019 data, counties in Coastal Kenya (Coast Endemic Zone) and western Kenya (Lake Endemic Zone) have a disproportionately high burden of malaria prevalence. A previous study by Dida et al*.* in 2018 found that western Kenya, or the Lake Endemic Zone, was the most affected area, with high levels of malaria and *Anopheles* mosquito prevalence [14]. Likewise, the Coast Endemic Zone had a high prevalence of malaria, with Kilifi and Kwale counties leading the region. A six-year surveillance study conducted by Dida et al*.* between 2003 and 2009 found that the Kenyan coast (Coast Endemic) accounted for 22% of paediatric admissions due to malaria, consistent with similar trends [14, 27]. These admission trends indicate a positive correlation between the prevalence of malaria parasites and mosquito species.

The prevalence of malaria is influenced by the distribution of *Anopheles* mosquitoes and specific climatic conditions. According to Ogola et al*.*, areas with high malaria rates have a higher prevalence of *An. funestus* compared to areas with low malaria rates [27]; the study further reveals that in 2020, the Coast Endemic region had a higher abundance of *An. funestus*. However, Karungu et al*.* reported different findings, with *An. gambiae* and *An. arabiensis* is the predominant malaria vector species in the coastal region [28]. Kipruto et al*.* [29] reiterate that temperatures between 16 °C and 34 °C are favourable for accelerating malaria vector metabolism, egg production, and blood meals, with the optimal temperature being 25 °C. Heavy rainfall and water stagnation also provide favourable breeding environments for malaria vectors [25]. In this study, these conditions were more apparent in malaria-endemic areas of the Lake Endemic and Coast Endemic areas, including areas with irrigation systems and along rivers. The study by Aregawi et *al.,* in Uganda, showed that *An. funestus* was the dominant malaria vector following the switch to a clothianidin-based indoor residual spraying (IRS) formulation, which explains the adaptive nature of the malaria vectors [11]. There were increased cases of malaria despite intensive mosquito control, underscoring the need to consider environmental factors, such as rainfall and water stagnation, that contribute to vector breeding in malaria control strategies [30]. The research findings emphasize that while mosquito surveillance is crucial, understanding malaria transmission requires a comprehensive approach. Integrating ecological data (e.g., rainfall, vegetation, elevation) with public health indicators (e.g., ITN coverage, access to treatment) is essential for designing effective, locally tailored interventions.

The study revealed significant associations among malaria prevalence, mosquito species, temperature, vegetation cover, growing season, animal density, and distribution of treated mosquito nets. Similar findings were reported in a study by Le et al*.* (2019), which showed that temperature and vegetation cover increase the risk of malaria in coastal Kenya [31]. These findings on the link between malaria prevalence, mosquito species, and temperature are consistent with those of a similar study in Zambia [32]. The results of this study are consistent with earlier research, highlighting the importance of accounting for local ecological conditions when developing targeted malaria control strategies and supporting the link between environmental factors such as temperature and vegetation cover and the elevated risk of malaria.

The findings of this study also suggest that aridity, elevation, and enhanced vegetation index could play a crucial role in predicting malaria prevalence in specific years. However, the non-significant coefficients for some independent variables suggest that other factors may be more critical in determining malaria prevalence in certain situations. These results are consistent with previous studies that have shown the complex interplay of various environmental, socio-economic, and cultural factors in shaping malaria transmission dynamics across different settings [28, 29].

The co-occurrence of both species in a region significantly elevates malaria transmission risk, with the Semi-Arid Seasonal zone posing a major concern due to the high density of *An. funestus*, a known efficient indoor biting and long-lived vector.

Environmental factors further influence mosquito distribution and malaria risk. *An. gambiae* prefers temporary, sunlit water pools that form after rainfall and shows adaptability to diverse habitats, including peri-urban areas. In contrast, *An. funestus* requires permanent, vegetated water bodies and is more dependent on consistent moisture from rainfall or irrigation. Temperature plays a crucial role for both species, with an optimal range between 20–30 °C. However, frost days and high elevations above 1800 m significantly limit mosquito survival, particularly affecting *An. funestus*. Vegetation-dense areas, especially those near homes with poor structural conditions (e.g., thatched roofs, open eaves), create ideal resting environments for *An. funestus*, which prefers indoor settings. *An. gambiae*, while often found indoors, is more flexible in its resting and breeding preferences. Additionally, high population density amplifies the risk of malaria transmission by increasing human-vector contact. The study’s findings emphasize the need for region-specific vector control strategies. Integrating mosquito surveillance with environmental and socio-economic data such as rainfall, housing quality, elevation, and population density can help design more effective interventions. Targeted approaches are especially important in high-risk zones like the Semi-Arid Seasonal areas, where vector density and environmental suitability align to sustain intense malaria transmission.

Malaria control efforts in Kenya align with the global plan for strategic vector elimination, which requires a comprehensive understanding of malaria vector distribution. Ogola et al*.* (2018) showed that *An. funestus* is a prominent vector in malaria transmission in Kenya, with higher prevalence in high-malaria zones [28]. The prevalence of malaria in different epidemiological zones depends on the mosquito species present, which is affected by different climatic conditions. The abundance of *An. gambiae* and *An. funestus* varies with changing climatic conditions, as observed in previous studies. For instance, a study in Uganda found that *An. gambiae* abundance varied seasonally, with peaks during the rainy season [33], while another study in Kenya found that *An. funestus* abundance was highest during the rainy season [5]. The study showed that fluctuations in the abundance of these species indicate that malaria transmission was ongoing throughout the analysed period. The seasonal variation in their abundance underscores the importance of implementing targeted interventions during high-risk periods to curb malaria transmission. Analysing the spatial distribution of mosquito species and their seasonal abundance can offer valuable insights into designing effective control measures to combat malaria transmission.

The study emphasizes the crucial roles of factors such as temperature and humidity, vegetation and water bodies, and human population density and mobility in maintaining malaria transmission. Furthermore, this study reports that higher malaria prevalence is associated with locations with a higher proportion of *An. funestus* than *An. gambiae,* such as the coastal areas. High-resolution mosquito sampling is essential to improve targeted control efforts, especially in light of recent malaria outbreaks in Uganda.

## Limitations

Consider these findings alongside their limitations. First, the spatial analysis of the relationship between mosquito species and malaria prevalence in Kenya’s epidemiological zones may have limited applicability for predicting outcomes at the village level due to insufficient data granularity. Moreover, the analysis results may not reveal significant patterns unless conducted at the village level. Additionally, potential bias from uneven sampling of mosquito species and a non-random sampling method may have compromised the analysis’s accuracy and reliability. Therefore, any conclusions drawn from this analysis should be interpreted with caution. Furthermore, the absence of longitudinal data across multiple years at the village level makes it difficult to accurately estimate the association between environmental factors, mosquito species distribution, and malaria prevalence over time.

## Conclusions

The study found significant differences in the spatial distribution of malaria in different sub-counties in Kenya. Climatic factors, such as increased rainfall and warm conditions, were associated with higher *Anopheles* mosquito density and subsequent malaria infections. *Anopheles funestus* was more commonly found in coastal areas with higher malaria transmission than *An. gambiae*. The study also emphasizes the significance of climatic factors in forecasting malaria vector distribution and incidence in several epidemiological zones of Kenya. The risk of malaria transmission increases when both *An. gambiae* and *An. funestus* are present, with the Semi-Arid Seasonal zone being of particular concern due to the high density of *An. funestus* there. Counties like Busia, Siaya, Kilifi, and Kwale in coastal Kenya, as well as the lake area, have disproportionately high malaria incidence rates. The prevalence of malaria is significantly influenced by temperature, plant cover, the growing season, animal population, and the spread of treated mosquito nets. These results can help guide malaria control efforts in Kenya and other nations, as they are consistent with research conducted in other African nations. Reducing the transmission of mosquito-borne diseases such as malaria requires targeted interventions and policies tailored to changing climate patterns.

## Supplementary Information

Supplementary material 1.

## Data Availability

The study data are available from the Demographic and Health Surveys (DHS) Program ([https://dhsprogram.com/] (https:/dhsprogram.com)) upon request. Entomological data can be obtained on request. Climatic and environmental data were sourced from the University of East Anglia Climatic Research Institute, following the guidelines described in the manuscript.

## Abbreviations

CRU: Climatic research unit
DHS: Demographic Health Survey
ELISA: Enzyme-linked immunosorbent assay
EVI: Enhanced vegetation index
GPS: Global positioning system
ITN: Insecticide treated nets
KEMRI: Kenya Medical Research Institute
LASSO: The least absolute shrinkage and selection operator
LLIN: Long-lasting insecticidal nets
NMCP: Kenya national malaria control programme
PCR: Polymerase chain reaction
WHO: World Health Organization
