# Supplementary material for: Mapping mosquito diversity in Kenya correlating species distribution with malaria prevalence across varied climatic parameters
Source: Malar J. 2026 Jan 5;25:76. doi: 10.1186/s12936-025-05713-y (PMC12870289; doi:10.1186/s12936-025-05713-y)
Supplement: Supplementary file 1 — Supplementary material 1. [file 12936_2025_5713_MOESM1_ESM.docx]

**Supplementary Material**

**S1: Table 4: Significant Climatic Predictors of Malaria Incidence in Kenya (2020) Identified via Lasso Regression.**


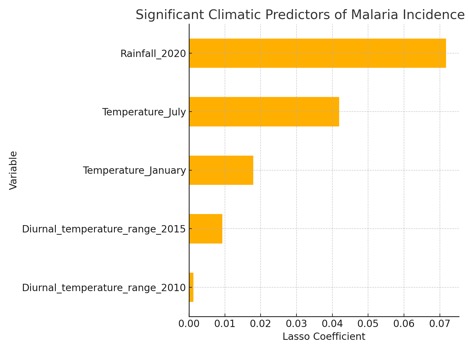


Lasso regression was used to isolate and evaluate climatic determinants of malaria incidence in Kenya in 2020. By restricting the analysis to environmental and meteorological variables, including temperature, rainfall, and aridity indices, the model efficiently selected the most influential predictors while minimizing the inclusion of spurious or collinear factors.

The analysis revealed that total rainfall in 2020 was the most prominent positive predictor of malaria incidence, corroborating existing evidence linking increased precipitation to the proliferation of mosquito breeding sites. Additionally, the mean temperature in July was positively associated with malaria incidence, likely reflecting the seasonal patterns that favour mosquito vector activity and parasite development during warmer periods.

Other contributing variables included wet days, diurnal temperature range, and specific monthly temperature means, underscoring the role of both cumulative and variability-driven climatic conditions in shaping malaria transmission dynamics.

These findings highlight emphasize the importance of incorporating high-resolution climate data into malaria surveillance, risk mapping, and early warning systems. They also provide valuable guidance for optimizing targeted vector control interventions in environmentally vulnerable regions.
